# Statin for mood and inflammation among adult patients with major depressive disorder: an updated meta-analysis

**DOI:** 10.3389/fpsyt.2023.1203444

**Published:** 2023-11-15

**Authors:** Xue Xiao, Hu Deng, Peng Li, Jifei Sun, Jing Tian

**Affiliations:** ^1^Department of Psychiatric, Beijing First Hospital of Integrated Chinese and Western Medicine, Beijing, China; ^2^Department of Innovation and Transformation, Beijing HuiLongGuan Hospital, Peking University HuiLongGuan Clinical Medical School, Beijing, China; ^3^The Key Laboratory of Geriatrics, Beijing Institute of Geriatrics, Institute of Geriatric Medicine, Chinese Academy of Medical Sciences, Beijing Hospital/National Center of Gerontology of National Health Commission, Beijing, China; ^4^Department of Radiology, Beijing First Hospital of Integrated Chinese and Western Medicine, Beijing, China

**Keywords:** depression, statin, mood, inflammation, meta-analysis

## Abstract

**Introduction:**

Several small sample-sized clinical trials have demonstrated a beneficial effect of statin on depressive mood among major depressive disorder (MDD) patients. However, observational studies have showed the increased risk of anxiety/depression with statin treatment. Therefore, we aimed to evaluate the effects of statin on depressive mood and inflammation status among MDD patients.

**Methods:**

We performed an updated meta-analysis RCTs identified in systematic searches of PubMed, Cochrane library, Embase, ClinicalTrials.gov, CNKI, Wan fang, VIP, and SinoMed database (up to August 2023). The primary endpoint was the Hamilton depression rating scale (HDRS). The secondary endpoints were rate of response to treatment, remission rate, levels of C-reactive protein (CRP), cognition and blood lipid. We evaluated the certainty of the evidence using the Grading of Recommendations Assessment, Development, and Evaluation (GRADE) approach.

**Results:**

The search identified seven RCTs involving 448 patients with a median follow-up of 10.4 weeks (range, 6–12 weeks). Compared with selective serotonin reuptake inhibitors (SSRIs) alone, treatment with statin plus SSRIs was associated with a significantly decreased HDRS [mean difference (MD) = −2.79; 95% confidence interval (CI): −3.83 to −1.76] and C-reactive protein (MD = −0.42 mg/L; 95% CI: −0.53 to −0.12 mg/L), and decreased levels of lipid profiles (*P* < 0.05). Moreover, statin plus SSRIs was associated with a comparable rate of treatment response [relative risk (RR) = 1.26; 95% CI: 0.98 to 1.62], remission rate (RR = 1.33; 95% CI: 0.89 to 1.99). Meta-regression indicated that the follow-up period was a source of heterogeneity regarding the HDRS (*r* = 0.302, *P* = 0.041). The quality of evidence was rated as moderate for HDRS and response rate according to the GRADE.

**Conclusion:**

Statin could safely and effectively improve the symptoms of depression and inflammation status among MDD patients.

**Systematic review registration:**

https://inplasy.com/inplasy-2022-3-0016/, identifier INPLASY2022230016.

## 1. Introduction

Depression contributes to a significant worldwide disease burden. It is the result of the interaction of complex social, psychological, and physiological factors, and in recent years, the neuroimmune hypothesis has become a research hotspot. In the past decade, it has been widely believed that inflammation is the driving force behind chronic fatal diseases, and increasing evidence suggests that immune disorders are related to depression (1). Although current research in the field of neuroimmunology mainly focuses on detecting systemic inflammation, there is still limited understanding of specific pathogenic pathways. A large clinical cohort study indicated that autoimmune diseases or serious infections would increase the risk of mood disorders later, and the younger the age of chronic inflammatory diseases, the higher the risk of depression (1). After the activation of innate immune cells, pro-inflammatory cytokines were produced, which acted on the central nervous system through the blood-brain barrier and cause pathological behavior (2). Proinflammatory cytokines can lead to depression by inhibiting monoamine neurotransmitters, activating the hypothalamic-pituitary-adrenal (HPA) axis, causing Th1/Th2 imbalance of helper T cell (Th) subsets, and influencing neurogenesis and plasticity (2). Therefore, immune inflammation can induce depression, and immunomodulatory therapy might contributes to antidepressant effects.

Clinical meta-analysis showed that anti-inflammatory drugs have antidepressant effects and do not increase the risk of gastrointestinal, cardiovascular events, and infections compared to placebo (3). The commonly used anti-inflammatory drugs currently include non-steroidal anti-inflammatory drugs (NSAIDs), cytokine inhibitors, antibiotics, etc. Among them, NSAIDs, statin, and minocycline are the most effective in alleviating depressive symptoms (3).

Clinical studies have shown that statin have auxiliary antidepressant effects. The antidepressant mechanisms include anti-inflammatory, antioxidant, neurotrophic, effects on monoamine neurotransmitters, and lipid-lowering function (4). The anti-inflammatory mechanism of statin is independent of their lipid-lowering effects, including reducing C-reactive protein (CRP) levels, indoleamine 2, 3-dioxygenase (IDO) activity, inhibiting the expression of pro-inflammatory cytokines on monocytes and lymphocytes. Besides, due to the high comorbidity rate between cardiovascular disease and depression, patients with comorbid cardiovascular disease can obtain additional antidepressant effects through statin therapy (5). Therefore, statins could be helpful for improving depressed mood among MDD patients (6–8).

In addition, several observational studies have showed that statin was associated with several mood/behavior changes (9, 10). Statin-associated mood/behavioral changes have been reported as psychiatric adverse drug reactions (ADRs) (10). Overall, although statin may only rarely cause ADRs, the potential adverse effects on mood/behavior warrant further investigation. Therefore, the effects of statin plus selective serotonin reuptake inhibitors (SSRIs) on mood and inflammation are still controversial, and we aimed to examine the efficacy of statin on depressive mood and inflammation status among MDD patients via a systematic review and meta-analysis of available RCTs. Furthermore, we evaluated the certainty of the evidence using the Grading of Recommendations Assessment, Development, and Evaluation (GRADE) approach.

## 2. Materials and methods

### 2.1. Data sources and search strategy

We conducted a literature search of the PubMed/MEDLINE, Cochrane Central Register of Controlled Trials (CENTRAL), Embase, ClinicalTrials.gov, CNKI, Wan fang, VIP and SinoMed database from the date of inception until August 2023. References of the included papers will be manually screened for further relevant material. We will contact the corresponding authors to obtain information about unpublished or incomplete trials. The review has been registered at https://inplasy.com/ (INPLASY2022230016).

The search strategy is {(“Hydroxymethylglutaryl-CoA Reductase Inhibitors” [MeSH]) OR *statin OR statins} AND (“Depression” [MeSH] OR “Depressive Disorder” [MeSH] OR “Depressive Disorder, Treatment-Resistant” [MeSH] OR “Depressive Disorder, Major” [MeSH] OR “Sleep” [MeSH] OR “Sleep Wake Disorders” [MeSH] OR “Sleep Initiation and Maintenance Disorders” [MeSH] OR “Sleep Stages” [MeSH] OR “Sleep, REM” [MeSH] OR “Sleep Disorders, Circadian Rhythm” [MeSH] OR “Anhedonia” [MeSH] OR “Anxiety” [MeSH] OR “Anxiety Disorders” [MeSH] OR “Psychomotor Disorders” [MeSH] OR depression OR depressive OR sleep OR insomnia OR sleep disorder OR anhedonia OR anxiety OR psychomotor retardation OR psychomotor impairment OR anx* OR antidepress*).

### 2.2. Study selection

First, we performed an initial screening of titles and abstracts. Second, all articles were evaluated based on full-text review. Studies were considered eligible if they met the following criteria: (1) adult patients (> 18 years), gender unlimited; (2) patients admitted to hospital and diagnosed as MDD according to Diagnostic and Statistical Manual of Mental Disorders, Fourth Edition (DSM-IV) criteria assessed at the screening visit, and the duration of MDD was at least 3 months; (3) without comorbid psychiatric disorder, or systemic disorder, such as hyperthyroidism or hypothyroidism, epilepsy, current active substance use, diabetes, hypertension, heart failure, myocardial infarction; (4) all were treated with a standard SSRI (citalopram; 40 mg/d) for at least 6 consecutive weeks; (5) those in the statin group were also given statin for at least 6 consecutive weeks; (6) primary endpoint was depression (HDRS score); (7) study design was a RCT; (8) sample size was > 20; (9) English or Chinese language articles. The exclusion criteria were: (1) patients had comorbid psychiatric disorder, systemic disorder, serious uncontrolled medical conditions, head trauma, intellectual disability, or family history of bipolar disorder (BD); (2) administrated with psychotic or antidepressant medication, electroconvulsive therapy; (3) with duplicated data.

### 2.3. Data extraction

Two reviewers (HD, PL) extracted the data on study characteristics (design, inclusion criteria, interventions, primary and secondary outcomes, quality), patient characteristics [sample size, age, percentage of males, percentage of current smokers, HDRS score, age at MDD onset, percentage of patients experiencing their first MDD episode, cognition, inflammation (CRP), duration of follow-up], interventions (type, dose, and duration of statin or SSRIs), lipid profiles, and study endpoints. The primary endpoint was depression (changes in HDRS). The secondary endpoints were the rate of response to treatment (with treatment response defined as a 50% decrease in the HDRS score), remission rate, cognition, inflammation (CRP) and changes in lipid levels. Rates of adverse events, such as myalgia and liver dysfunction, were also recorded. All disagreements among reviewers were resolved by discussion with another reviewer (XX).

### 2.4. Quality evaluation

The preferred reporting items for systemic reviews and meta-analyses statement was followed to perform the quality evaluation (11). Using the Cochranes risk of bias tool, two reviewers (JS and JT) evaluated the quality of included studies, including the generation of random sequences, allocation concealment, blinding of participants and personnel, blinding of outcome assessment, incomplete outcome data, selective reporting, and other bias.

We used the GRADEpro GDT software to evaluate the certainty of evidence according to the GRADE guidelines for HDRS and rate of response based on areas of study design, risk of bias, inconsistency, indirectness, imprecision, and other considerations, such as publication bias, effect size, and potential confounding (12).

According to GRADE Working Group, grades of evidence were divided into (1) high quality: we are very confidence that the true effect lies close to that of the estimate of the effect; (2) moderate quality: we are moderately confident in the effect estimate: the true effect is likely to be close to the estimate of the effect, but there is a possibility that it is substantially different; (3) low quality: our confidence in the effect estimate is limited: the true effect may be substantially different from the estimate of the effect; (4) very low quality: we have every little confidence in the effect estimate: the true effect is likely to be substantially different from the estimate of effect.

### 2.5. Data analysis

The results were analyzed with Stata 16.0 (CA, USA). Based on the level of heterogeneity (13), we used the fixed-effects model or random-effects model to evaluate the outcomes. The relative risk (RR) was calculated for dichotomous variables, while the mean difference (MD) was calculated for continuous variables.

We used the *I*^2^ statistic and the Q test to assess the levels of heterogeneity. *I*^2^ > 50% was indicated a significant heterogeneity (14), necessitating the use of the random-effects model; all data with non-significant heterogeneity (*I*^2^ ≤ 50%) were analyzed using the fixed-effects model. If there was significant heterogeneity, between-study sources of heterogeneity were assessed using subgroup analyses. Besides, the publication bias was evaluated with Egger’s regression test (*P* ≤ 0.10) (15) and funnel plots (16). Sensitivity analyses were also performed to determine the effect of an individual study on the overall outcome. The statistical significance level was set as 0.05.

### 2.6. Meta-regression

Univariate meta-regression analysis was used to identify possible contributors to between-study variance. We investigated the associations between the SMD of the HDRS and clinically plausible factors, including sample size, age, percentage of males, educational level, percentage of current smokers, HDRS, age at MDD onset, CRP level, percentage of patients experiencing their first MDD episode, cognition, lipid profiles (TC, LDL, HDL, and TG levels), and duration of follow-up.

### 2.7. Subgroup analyses

Based on the baseline condition of the patients (MDD vs. MDD or BD), the studies were divided into “MDD” and “MDD or BD” subgroups. In accordance with baseline clinical factors, all studies were classified into subgroups based on sample size (< 50 or ≥ 50), age (< 39.6 years or ≥ 39.6 years), proportion of males (< 42.8 or ≥ 42.8%), educational level of primary school or above (< 76.9 or ≥ 76.9%), proportion of current smokers (< 41.6 or ≥ 41.6%), level of HDRS (< 24.5 or ≥ 24.5), age at MDD onset (< 32.5 years or ≥ 32.5 years), proportion of patients experiencing their first MDD episode (< 56.5 or ≥ 56.5%), levels of serum TC (< 168.4 or ≥ 168.4 mg/dl), LDL-C (< 97.4 or ≥ 97.4 mg/dl), HDL-C (< 50.1 or ≥ 50.1 mg/dl), and TG (< 115.5 or ≥ 115.5 mg/dl). Additionally, studies were divided on the basis of follow-up duration into subgroups of < 3 weeks, 3–5 weeks, 6–11 weeks, and ≥ 12 weeks.

## 3. Results

### 3.1. Search results

**FIGURE 1:**
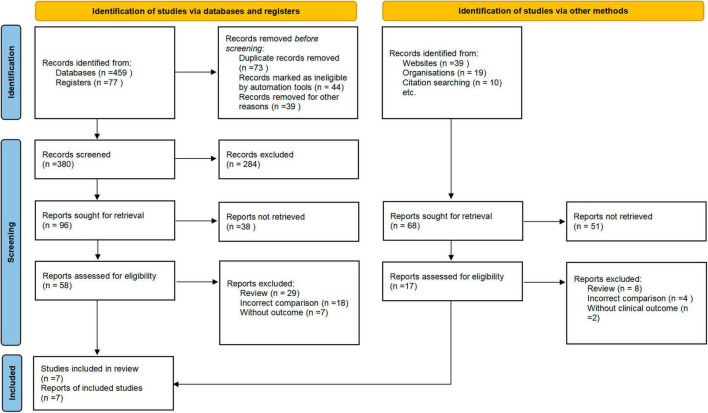
Flow diagram.

A total of 387 articles were initially identified after the screening of the titles and abstracts. Among them, 73 articles were retrieved for full-text review. After the exclusion of 66 articles that did not meet the eligibility criteria, a final total of seven RCTs were included (Figure 1).

### 3.2. Study’s characteristics

The study characteristics are summarized in Table 1. Of the seven included studies, all were completed and reported clinical outcomes (17–23). Six RCTs enrolled patients with MDD (17–20, 22, 23), while one enrolled patients with MDD or BD (21). One RCT enrolled patients with a history of coronary artery bypass surgery in the last 6 months (20). Six RCTs analyzed the effect of statin plus SSRIs versus SSRIs alone (17–19, 21–23), while one assessed the effect of simvastatin versus atorvastatin (20). Most studies administered simvastatin and atorvastatin for 6–12 weeks (18–21, 23). The primary endpoint was depression (indicated by HDRS score) in six RCTs (17–20, 22, 23).

**TABLE 1:** Baseline characteristics of clinical trials included.

Trial/References	Study design	Patients	Statins + SSRIs treatment group	SSRIs treatment group	Study endpoint
(17)	RCT	(1) Adult patients; (2) Diagnosed as MDD according to DSM-IV criteria; (3) Without serious uncontrolled medical conditions and positive family history for bipolar disorder	(1) Fluoxetine, up to 40 mg/d, for 6 weeks; (2) Lovastatin, 30 mg/d, for 6 weeks	(1) Fluoxetine, up to 40 mg/d, for 6 weeks; (2) Placebo, for 6 weeks	(1) Primary outcome: HDRS score change from baseline to week 6 between groups; (2) Adverse effects, muscle pain, etc.
(18)	RCT	(1) Aged 18–50 years; (2) Diagnosed as unipolar depressive disorder according to DSM 5 or HDRS ≥ 25 at the screening visit; (3) Without comorbid psychiatric disorder, or systemic disorder	(1) Citalopram, 40 mg/d, for 12 weeks; (2) Atorvastatin, 20 mg/d, for 12 weeks	(1) Citalopram, 40 mg/d, for 12 weeks; (2) Placebo, for 12 weeks	(1) Primary outcome: HDRS score change from baseline to week 6 between groups; (2) Secondary outcome: remission rate, response rate, change of blood lipid (3) Adverse event: muscle pain, etc.
(19)	RCT	(1) Aged 20–70 years; (2) Diagnosed as MDD based on DSM IV-TR criteria (3) With a HDRS score ≥ 22 at the screening visit	(1) Fluoxetine, up to 20 mg/d, for the first 2 weeks; followed by 40 mg/d for 4 weeks (2) Simvastatin, 20 mg/d, for 6 weeks	(1) Fluoxetine, up to 20 mg/d, for the first 2 weeks; followed by 40 mg/d for 4 weeks	(1) Primary outcome: HDRS score change from baseline to week 6 between groups; (2) Secondary outcome: early improvement, response rate and remission rate (3) Adverse event: muscle pain, liver function, etc.
(20)	RCT	(1) Aged > 18–50 years; (2) With a history of CABG in the last 6 months; (3) Diagnosed as MDD based on DSM IV-TR criteria	(1) Simvastatin, 20 mg/d, for 6 weeks	(1) Atorvastatin, 20 mg/d, for 6 weeks	(1) Primary outcome: simvastatin efficacy in improvement of HDRS score; (2) Secondary outcomes: change of HDRS score and time needed to respond to treatment; (3) Adverse event: muscle pain, etc.
(21)	RCT	(1) Aged > 18–85 years; (2) Diagnosed as BD (*n* = 54) and MDD (*n* = 6); (3) Taking lithium and had nephrogenic diabetes insipidus	(1) Standard SSRIs therapy, for 12 weeks (2) Atorvastatin, 20 mg/d, for 12 weeks	(1) Standard SSRIs therapy, for 12 weeks (2) Placebo, for 12 weeks	(1) Primary outcome: cognitive function, global cognition Z-score assessed at 12-week follow-up (2) Secondary outcome: mood performance, depression relapse at either 4- or 12-week follow up (3) Adverse event: muscle pain, etc.
(22)	RCT	(1) Aged 23–49 years; (2) Diagnosed as MDD; (3) Without axis *I* mental conditions or addictions, or head trauma, or chronic medical disease	(1) Standard SSRIs therapy, for 12 weeks (2) Rosuvastatin, 10 mg/d, for 12 weeks	(1) Standard SSRIs therapy, for 12 weeks (2) Placebo, for 12 weeks	(1) Primary outcome: HDRS score and BDI score assessed at 3 month (2) Secondary outcome: brain perfusion and neurocognitive performance (3) Adverse event: muscle pain, etc.
(23)	RCT	(1) Adults, aged 18–75 years; (2) Diagnosed as treatment resistant MDD; (3) Without primary psychotic disorder or bipolar disorder, or history of intolerance to statins or any unstable physical condition or neurological problem	(1) Standard SSRIs therapy, for 12 weeks (2) Simvastatin, 20 mg/d, for 12 weeks	(1) Standard SSRIs therapy, for 12 weeks (2) Placebo, for 12 weeks	(1) Primary outcome: Montgomery-Asberg Depression Rating Scale (MADRS) 27 scores at week 12; (2) Secondary outcome: rates of response and remission, with response defined as 50% or greater reduction in MADRS scores and remission defined as a MADRS score of 10 or less at week 12. (3) Adverse event: muscle pain, etc.

RCT, randomized clinical trial; SSRI, selective serotonin reuptake inhibitor; MDD, major depressive disorder; CABG, coronary artery bypass surgery; BD, bipolar disorder; MADRS, Montgomery-Asberg depression rating scale; DSM-IV, Diagnostic and Statistical Manual of Mental Disorders, Fourth Edition; HDRS, Hamilton depression rating scale; BDI: Beck’s depression inventory.

### 3.3. Patient’ characteristics

The seven RCTs included a total of 448 patients (17–23). The patient characteristics are summarized in Table 2. The number of patients in each RCT was 20–150, and the median duration of follow-up was 10.4 weeks (range 6–12 weeks). The mean participant age was 39.4 years. Nearly half (41.4%) of the patients were men. Most patients (76.9%) had an educational level of primary school or above, and 37.4% were current smokers. The baseline mean HDRS score was 26.8. The mean age at MDD onset was 32.5 years, and 56.5% of patients were experiencing their first MDD episode. Only two RCTs reported baseline cognition (21, 22). The mean levels of TC, LDL, HDL, and TG were 168.4, 97.4, 50.1, and 115.5 mg/dl, respectively.

**TABLE 2:** Baseline characteristics of patients in the statin + SSRIs and SSRIs therapy groups.

References	Year	Patient’s Num.	Age, year	Male, %	Education, year	Smoking, %	Baseline HDRS	MDD onset, age	First MDD episode, %	Baseline cognition	CRP,ug/mL	Follow, week
(17)	2013	34/34	32.5/31.7	35.3/38.2	NR	NR	28.9/27.6	NR	NR	NR	NR	6
(18)	2014	30/30	33.1/31.4	53.3/53.3	100/100@	NR	33.3/34.3	NR	2.3/1.99ε	NR	NR	12
(19)	2015	22/22	36.4/34.2	41.0/27.0	81.0/68.0Φ	27.0/18.0	23.6/25.0	35.1/32.5	63.0/59.0	NR	NR	6
(20)	2015	23/23	56.4/57.7	69.6/65.2	30.4/34.8Φ	47.8/39.1	15.9/16.1	NR	NR	NR	NR	6
(21)	2020	27/33	47.8/53.1	51.9/33.3	96.3/100§	NR	4.2/4.5#	NR	4.9/5.0ε	0.015/−0.026#	NR	12
(22)	2022	10/10	31.5/36.5	30.0/30.0	17/17	50.0/30.0	21.0/23.0	30/33	50/50	CANTAB test	0.93/0.32	12
(23)	2023	77/73	40.0/35.0	44.0/45.0	8.0/8.0	13.0/16.0	30.0/31.0	NR	NR	NR	1.39/1.39	12

#, Montgomery–Asberg depression rating scale; @, percentage of education ≥ primary school; Φ, percentage of education ≥ diploma; §, percentage of education ≥ high school; #, global cognition Z-score; ε, number of post mood episodes; HDRS, Hamilton depression rating scale; MDD, major depressive disorder; CANTAB, Cambridge neuropsychological test automated battery; Num, number; NR, not reported.

### 3.4. Methodological quality assessment

**FIGURE 2:**
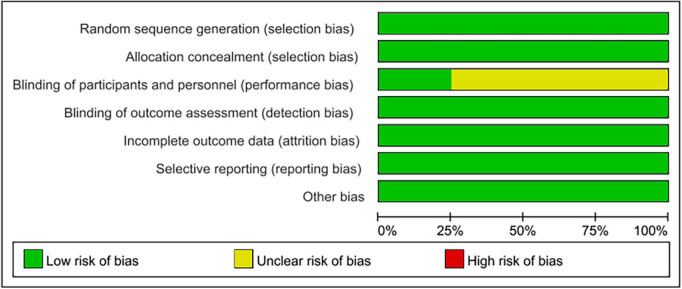
Quality evaluation with Cochrane’s risk of bias tool.

Seven RCTs randomized the participants and reported the details of random sequence generation (17–23). All RCTs used concealed treatment allocation methods (17–23). Four RCTs reported the methods used to blind participants and personnel (19–21, 23), and six RCTs reported the methods used for blinding of outcome assessments (17–20, 22, 23). The attrition bias and selective reporting bias were low in all except one RCT (21) (Figure 2).

### 3.5. Primary endpoint

#### 3.5.1. Depression

**FIGURE 3:**
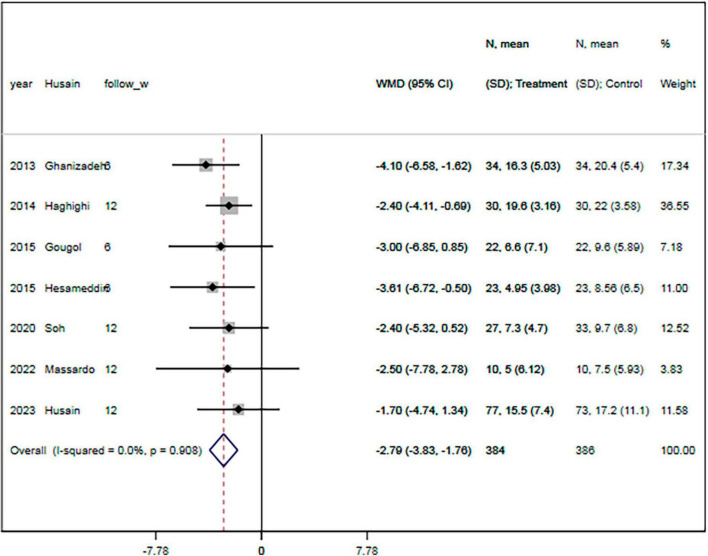
Statin treatment was associated with decreased HDRS. Fixed-effect model (*I*^2^ = 0.0%). HDRS, Hamilton depression rating scale; MD, mean difference; CI, confidence interval.

All seven RCTs provided data on the depressive symptoms evaluated by changes of HDRS score (17–23). There were 223 and 225 patients in the statin plus SSRIs and SSRIs alone groups, respectively. The statin plus SSRIs group had a significantly lower HDRS score than the SSRIs alone group (MD = −2.79; 95% CI: −3.83 to −1.76; *P* < 0.001) (Figure 3); however, there was a high level of heterogeneity (*I*^2^ = 0.0%). Sensitivity analysis indicated that the removal of any single study had no significant effect on the HDRS result.

### 3.6. Secondary endpoints

#### 3.6.1. Treatment response rate

**FIGURE 4:**
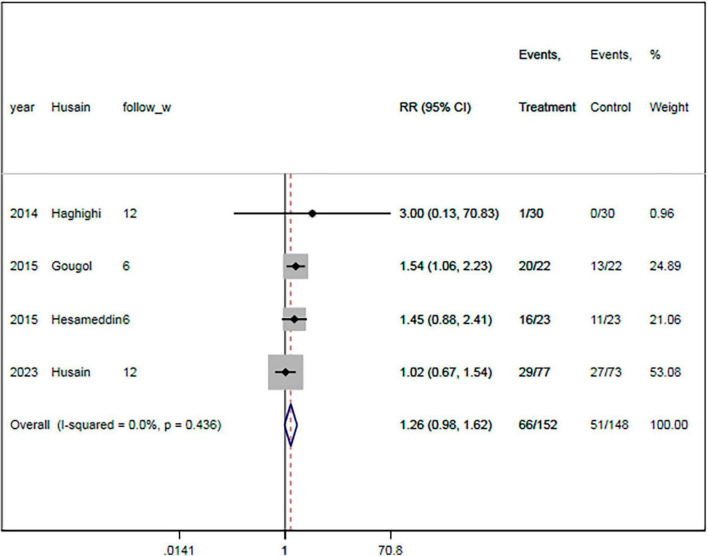
Statin treatment was associated with similar rate of respond to therapy. Fixed-effect model (*I*^2^ = 0.0%). RR, relative risk; CI, confidence interval.

The rate of response to treatment was specified in four RCTs (18–20, 23). There were 152 and 148 patients in the statin plus SSRIs and the SSRIs alone groups, respectively. The response to treatment was shown for 66 patients in the statin plus SSRIs group and 51 in the SSRIs alone group. Overall, the statin plus SSRIs group was associated with a comparable rate of treatment response compared with the SSRIs alone group (RR = 1.26; 95% CI: 0.98 to 1.62; *P* = 0.077) (Figure 4). There was a low level of heterogeneity (*I*^2^ = 0.0%).

### 3.7. Remission rate

**FIGURE 5:**
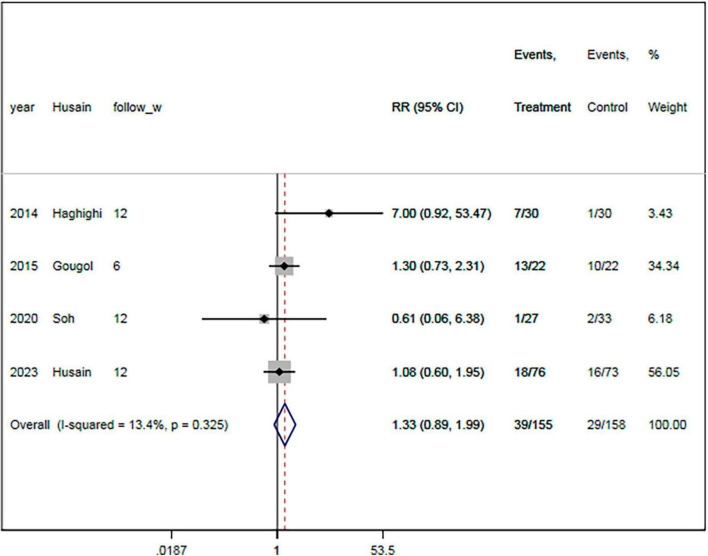
Statin treatment was linked with comparable remission rate. Fixed-effect model (*I*^2^ = 20.7%). RR, relative risk; CI, confidence interval.

The remission rate was reported in four RCTs (18, 19, 21, 23). There were 155 and 158 patients in the statin plus SSRIs and the SSRIs alone groups, respectively. A total of 39 patients in the statin plus SSRIs group and 29 in the SSRIs alone group went into remission during the follow-up period. Overall, treatment with statin plus SSRIs achieved a similar remission rate to SSRIs alone (RR = 1.33; 95% CI: 0.89 to 1.99; *P* = 0.166) (Figure 5). There was a low level of heterogeneity (*I*^2^ = 20.7%).

### 3.8. Level of CRP

Only one RCT reported CRP level (22). Compared with the SSRIs alone group, the statin plus SSRIs group had a significantly decreased level of high-sensitive C-reactive protein (MD = −0.42 mg/L; 95% CI: −0.53 to −0.12 mg/L; *P* = 0.015). There was a low level of heterogeneity (*I*^2^ = 0%).

### 3.9. Cognition

Only two RCTs reported cognition (21, 22). Compared with the SSRIs alone group, the statin plus SSRIs group had a similar global cognition Z-score (MD = −0.16; 95% CI: −0.65 to 0.34; *P* = 0.620). One study showed important improvements in regional blood flow and neurocognitive performance assessed by functional MRI (*P* < 0.05) (22).

### 3.10. Lipid profiles

Three RCTs reported the lipid levels (18, 20, 22). Overall, the administration of statin plus SSRIs was associated with significantly decreased levels of TC (MD = −1.76; 95% CI: −3.48 to −0.27; *P* = 0.013; *I*^2^ = 91.1%), LDL (MD = −1.73; 95% CI: −3.35 to −0.29; *P* = 0.030; *I*^2^ = 90.9%), and TG (MD = −0.64; 95% CI: −0.83 to −0.11; *P* = 0.003; *I*^2^ = 0.0%) compared with the control group. The HDL level was significantly increased in the statin plus SSRIs group compared with the SSRIs alone group (MD = 0.79; 95% CI: 0.23 to 0.99; *P* = 0.003; *I*^2^ = 33.7%).

### 3.11. Adverse events

The rate of adverse events was specified in six RCTs (17–20, 22, 23). Table 3 summarizes the reported adverse events. Most RCTs did not report the occurrence of myalgia or liver dysfunction. One RCT reported nine adverse events, including myalgia, increased appetite, decreased appetite, nausea, vomiting, headache, constipation, insomnia, and abdominal pain (19). Another RCT reported that one patient developed asymptomatic mild elevation of hepatic enzymes (22).

**TABLE 3:** Rate of major adverse event.

References/ RCTs		Myalgia	Liver dysfunction	Nausea	Vomiting	Decreased appetite
(17)	Statins + SSRIs group	0/34	0/34	16/34	4/34	1/34
	SSRIs group	0/34	0/34	16/34	4/34	8/34
(18)	Statins + SSRIs group	0/30	NR	NR	NR	NR
	SSRIs group	0/30	NR	NR	NR	NR
(19)	Statins + SSRIs group	9/44§	0/22	9/44§		
	SSRIs group		0/22			
(20)	Statins + SSRIs group	0/23	0/23	0/23	0/23	2/23
	SSRIs group	0/23	0/23	0/23	0/23	0/23
(21)	Statins + SSRIs group	NR	NR	NR	NR	NR
	SSRIs group	NR	NR	NR	NR	NR
(22)	Statins + SSRIs group	0/10	1/20#	NR	NR	NR
	SSRIs group	0/10		NR	NR	NR
(23)	Statins + SSRIs group	2/77	NR	5/77	NR	2/77
	SSRIs group	0/73	NR	3/73	NR	0/73

§, nine adverse event were reported in patients, including myalgia, increased appetite, decreased appetite, nausea, vomiting, headache, constipation, insomnia, and abdominal pain; #,one with asymptomatic mild elevation of hepatic enzymes; RCT, randomized controlled trial; SSRI, selective serotonin reuptake inhibitor.

### 3.12. Meta-regression analysis

**FIGURE 6:**
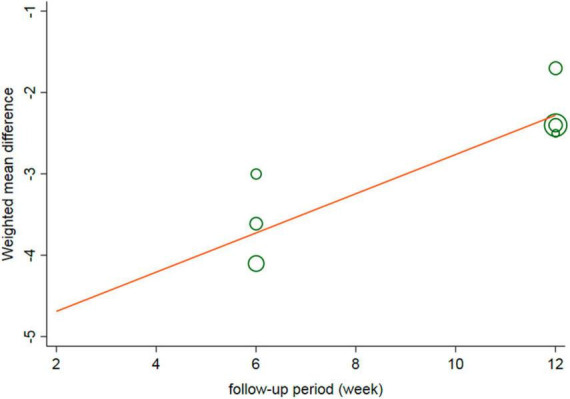
Meta-regression of change of HDRS and follow-up period. HDRS, Hamilton depression rating scale; SMD, standard mean difference; CI, confidence interval.

The meta-regression analysis results showed no significant correlations were observed between the MD of the HDRS and the condition of the patients (*P* = 0.434), sample size (*P* = 0.764), age (*P* = 0.497), proportion of males (*P* = 0.919), educational level (*P* = 0.585), smoking status (*P* = 0.818), baseline HDRS (*P* = 0.625), age at MDD onset (*P* = 0.193), proportion of patients experiencing their first MDD episode (*P* = 0.718), and baseline levels of TC (*P* = 0.718), LDL (*P* = 0.753), HDL (*P* = 0.626), and TG (*P* = 0.856). However, the follow-up period was positively associated with the SMD of the HDRS (*r* = 0.302, *P* = 0.041) (Figure 6).

### 3.13. Subgroup analyses

**FIGURE 7:**
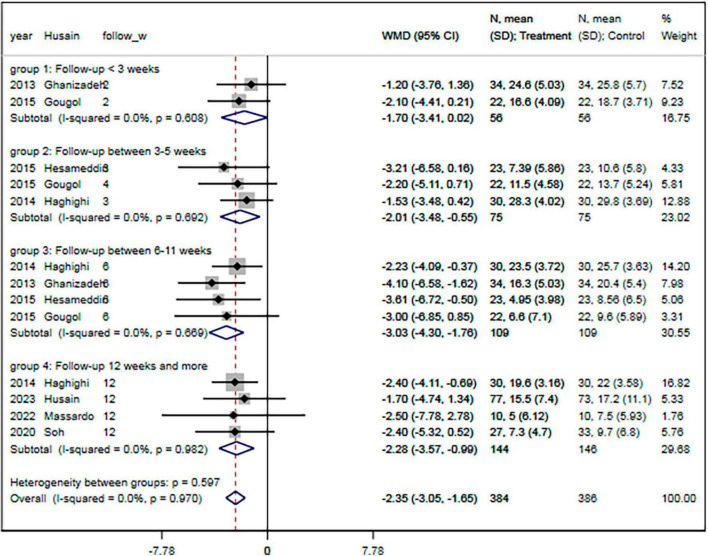
Subgroup analysis of change of HDRS with different follow-up period. HDRS, Hamilton depression rating scale; SMD, standard mean difference; CI, confidence interval.

The subgroup analyses results showed that the pooled MDs of the HDRS were significantly lower in the statin plus SSRIs group than in the SSRIs alone group in studies with a follow-up duration of 3–5 weeks (MD = −2.01; 95% CI: −3.48 to −0.55) or 6–11 weeks (MD = −3.03; 95% CI: −4.30 to −1.76) or ≥ 12 weeks (MD = −2.28; 95% CI: −3.57 to −0.99) (Figure 7). In addition, there were significant differences in the HDRS among subgroups based on patient condition, sample size, age, proportion of males, educational level, baseline HDRS, proportion of patients experiencing their first MDD episode, and levels of TC, LDL, and HDL (all *P* < 0.05).

### 3.14. Grading of evidence

The GRADE protocol was used to assess the certainty of the evidence (Table 4). Accordingly, studies investigating the effect of statins on HDRS and rate of response were regarded as moderate quality, due to the low heterogeneity between studies or relatively small sample size.

**TABLE 4:** GRADE profile of statins for HDRS, rate of response, and remission rate.

Quality assessment							No. of patients		Effect	Quality
No. of studies	Design	Risk of bias	Inconsistency	Indirectness	Imprecision	Other considerations	Statins	Control	MD/RR (95% CI)	
**HDRS (follow-up 6–12 weeks; better indicated by lower values)**										
7	Randomized trials	No serious risk of bias	Serious^1^	No serious indirectness	No serious imprecision	None	223	‘225	–2.79 (-3.83, –1.76)^4^	MODER-ATE
**Rate of response (follow-up 6–12 weeks; better indicated by higher values)**										
4	Randomized trials	No serious risk of bias	No serious^2^	No serious indirectness	No serious imprecision	None	152	148	1.26 (0.98, 1.62)	MODER-ATE
**Remission rate (follow-up 6–12 weeks; better indicated by lower values)**										
4	Randomized trials	No serious risk of bias	No serious^3^	No serious indirectness	No serious imprecision	None	155	158	1.33 (0.89, 1.99)	LOW

^1^The test for heterogeneity is not significant, and the *I*^2^ is high, 0.0%. ^2^The test for heterogeneity is not significant, and the *I*^2^ is low, *I*^2^ = 0.0% ^3^The test for heterogeneity is not significant, and the *I*^2^ is low, *I*^2^ = 20.7% ^4^Effect is the mean difference and 95% CI. HDRS, Hamilton depression rating scale; MD, mean difference; RR, relative risk; CI, confidence interval.

## 4. Discussion

Our updated meta-analysis and systematic review of six RCTs showed that when used as add-on treatment to SSRIs, statin significantly decreased the severity of depressive symptoms. Furthermore, it was associated with an increased treatment response rate and decreased hs-CRP levels. Myalgia and liver dysfunction were rare and not severe. Meta-regression indicated that the duration of follow-up was one of the sources of heterogeneity regarding the HDRS (*r* = 0.425, *P* = 0.034). Subgroup analysis showed that the HDRS was only decreased in studies with a medium follow-up duration. The GRADE protocol showed that studies investigating the effect of statins on HDRS and rate of response were regarded as moderate quality. Overall, statins appear to provide a safe clinical effect in improving depressive symptoms in patients with MDD.

Statins might have the a preventive effect against the occurrence of depression among patients with hyperlipidemia. Recently, a nationwide cohort study that compared incident SSRI alone users (*n* = 872,216) versus those administered statin with an SSRI (*n* = 113,108) indicated that the administration of statin plus an SSRI significantly lowered the risk of first onset of depression compared with an SSRI alone (21). Moreover, several observational studies have indicated that statin had a preventive effect against the occurrence of depression in patients with hyperlipidemia (22). A meta-analysis of seven observational studies showed that statin users had a 32% lower risk of developing depression than non-statin users (24). However, other studies have indicated that long-term statin therapy increased the risk of depression development. A prospective cohort study of 1,631 subjects indicated that regular statin use dids not lower the risk of depression during a 5.2 years follow-up (25). Therefore, more studies are needed to confirm the long-term effect of statins on depression occurrence.

Statins could be effective adjunctive treatments for depression mood among MDD patients. A randomized trial showed that the administration of fluoxetine combined with simvastatin results in significantly greater improvements in depressive symptoms than fluoxetine used alone (19). Another clinical trial found that atorvastatin enhances the effect of citalopram in the treatment of MDD (18); compared with the single use of citalopram, the HDRS score was significantly reduced after 12 weeks of combined medication (18). Furthermore, 6 weeks of treatment comprising fluoxetine combined with atorvastatin is more effective than fluoxetine monotherapy in reducing the clinical symptoms of moderate and severe depression (evaluated using the HAMD score) without increasing ADRs (26), and the addition of lipid-lowering drugs significantly reduces HAMD scores compared with SSRIs alone (6). In a retrospective cohort study involving 13,626 statin users from the US military health-care system, the authors found that compared with statin persistent users, statin non-persistent users were associated with higher increased risk schizophrenia/psychosis (OR = 1.58, 95% CI: 1.20–2.10). Similarly, we confirmed that statins improved the depressive symptoms and treatment response rate. Meanwhile, they also found that statins were associated with decreased risk of cognitive disorders. Therefore, they concluded that cumulative persistent with statin exposure was associated with lower prevalence of cognitive decline and diagnosis of mood disorders (27). Besides, one study showed important improvements in regional blood flow and neurocognitive performance assessed by functional MRI (*P* < 0.05) (22). However, in the RCT conducted by Fotso Soh et al. (21), they found that atorvastatin and placebo groups did not differ in terms of global cognition Z-score after 12 weeks follow-up. Overall, these studies show that the effects of statin plus SSRIs on mood and cognition are still controversial.

However, several case reports have detected an increased severity of depressive symptoms and cognitive decline with statin treatment (8). Statin-associated mood/behavioral changes have been reported as psychiatric adverse drug reactions (ADRs). A case series reported that 12 patients had ADRs comprising mood/behavioral changes that commenced following the initiation of statin and persisted or progressed with continued statin use (9). Another case series included 6 patients with ADRs and reported that severe irritability might occur in some statin users (10). Among 329 elderly (≥ 65 years) patients from an population-based study, the authors found that statin users were much more likely to suffer of cognition decline and depression than statin non-users, characterized by lower mini mental state examination score (MMSE) and higher geriatric depression scale (GDS) score (28). This finding was confirmed by a prospective, open-level study, which further indicated an improvement in cognition with discontinuation of statin use and worsening cognition with continuation of statin use (29). These observations suggested that mood and behavioral changes might occur in some patients taking statins, though a recent systematic review of 72 studies indicated that statin treatment was unlikely to lead to depressive symptoms in the general population (30). Overall, although statin may only rarely cause ADRs, the potential adverse effects on mood/behavior warrant further investigation.

The mechanism of statin against depression is still controversial. Initial studies suggested that statin might improve the quality of life by reducing cardiovascular risk, thereby relieving the symptoms of MDD. In recent years, statin have also been used to stabilize plaque, improve blood supply to the infarcted area, and inhibit local functional cell apoptosis, thereby improving serotonin and norepinephrine neuronal pathways among patients with post-stroke depression (31). As high levels of cholesterol increase the risk of MDD, statin may inhibit the occurrence of MDD by lowering cholesterol. Patients with depression have higher levels of pro-inflammatory markers, which are lowered by the anti-inflammatory and antioxidant effects of statin treatment (32). Furthermore, statins decrease the enzyme activity of indoleamine-2,3 dioxygenase, which converts tryptophan to neurotoxic compounds and leads to depression; thus, statins result in higher tryptophan and serotonin levels and decrease the development of depression (33). In our study, meta-regression showed no association between the HDRS and post-therapeutic or percentage changes in lipid levels. Therefore, the antidepressant effects of statin may be independent of the lipid-lowering mechanism. However, statin agents increase the cerebral perfusion and oxygenation through the reduction of vascular plaques, which might result in a lower risk of depression, especially among aging adults (34).

The effect of statin in improving depressive symptoms in patients with MDD can be explained from several pathophysiological mechanisms (35). First, an animal study using a mouse model showed that the antidepressant effect of statin may be attained through the L-arginine-nitric oxide-acycloguanosine monophosphate pathway. Atorvastatin inhibited N-methyl-D-aspartate receptors and nitric oxide-acycloguanosine monophosphate phosphate synthesis, leading to downregulation of excitatory processes. At the behavioral level, this downregulation may reflect a reduction in depressive symptoms. Second, the acute effect of statin may also increase the release of brain-derived neurotrophic factors, and an increased level of brain-derived neurotrophic factors was associated with improvements in depressive symptoms. Third, depression was associated with impaired immune function, increased immune activation, and increased oxidative stress. Statins could inhibit inflammatory cytokines and reduce markers of oxidative stress (36). A previous meta-analysis showed that the level of peroxides in the blood lipids was higher in patients with depression than in those without depression and was related to the severity of depression, while antidepressant treatment reduces peroxide markers. Meanwhile, statin also reduced depression-related pro-inflammatory cytokine markers such as hs-CRP (36). Several previous studies have showed implicating inflammatory mechanisms in depression. Alterations in the cytokine system as well as higher levels of hs-CRP have been shown in depression patients. Therefore, these mentioned data along with the anti-inflammatory potential of statin further support the benefits for depression.

Patients with MDD may benefit from statin therapy. Several studies have indicated that a higher CRP level might predict better statin treatment effects. Therefore, patients with depression with elevated inflammatory markers might benefit from agents with anti-inflammatory properties (37). In addition, there is some evidence that the blood-brain barrier (BBB) permeability might affect the antidepressant effects of statin treatment (38). As simvastatin has greater lipophilic properties, it crosses the BBB more easily than other statin. A head-to-head comparison study showed that simvastatin is associated with a better antidepressant effect than atorvastatin (20). However, the optimal dose of statin has not been evaluated. Furthermore, some studies have indicated that long-term statin therapy may result in depression, and that a very low LDL level may be a risk factor for depressive symptoms (6, 39). These issues warrant investigation in future studies.

Hyperlipidemia was a predictor of depression, and a high lipid level was linked to a faster increase in the HDRS (40). A study investigating the relationship among depressive symptoms, health behaviors, and blood lipid levels in young adult women showed that depression was moderately negatively associated with LDL level, and MDD was associated with low insolubility. Moreover, dietary fiber intake was related to both HDL and LDL levels. In unadjusted analyses, depression had an indirect effect on LDL level through dietary fiber intake (41, 42). Thus, depression was inversely associated with serum LDL levels in young adult women, but this relationship was not mediated by poor lifestyle. In our study, meta-regression analyses did not detect a significant association between the HDRS and the baseline LDL level.

Compared with several relevant systematic review and meta-analysis (3, 6, 24, 43–45), there were several strengths in our study. (1) We found that statin treatment significantly improved the treatment response rate, which was not reported in the two previous studies (6, 24). (2) Our study detected that statin therapy was safe in patients with depression, and the effect of statin in relieving depressive symptoms was independent of the baseline HDRS score or treatment duration (43–45). (3) Although statin were associated with significantly improved lipid levels, meta-regression showed that there was no association between the HDRS and post-therapeutic or percentage changes in lipid levels. (4) We also found that statins were associated with decreased level of CRP, which may be related to the anti-depressive effect of statins.

## 5. Study limitations

The present study has several limitations. (1) Our meta-analyses was based on study-level data with the flaws of the original RCTs. (2) The included studies used four different statin agents (lovastatin, simvastatin, atorvastatin, rosuvastatin) with different antidepressant and anxiolytic effects, which may have impacted the overall outcome of statin treatment. Simvastatin possesses the greatest ability to infiltrate the BBB, and the response rate is reportedly significantly higher in those taking a lipophilic statin agent (41). However, simvastatin was only used in two included studies. (3) There may be geographical variations in the study results. All six included studies had differences in patient characteristics, administered statin agents, and duration of follow-up. (4) The overall sample size was small. (5) The median follow-up was 9 weeks. However, longer follow-up is needed, as statin therapy is expected to accrue over time.

## 6. Conclusion

We found that statin could safely and effectively improved the severity of depression mood and decreased inflammatory status, but had no significant effect on cognition. However, this result must be interpreted with caution, for the evidence supporting the use of statin for MDD is limited by the small sample size and short follow-up duration of previous studies. Large RCTs are warranted to confirm the value of statin therapy in patients with MDD, especially among those with diseases of the cardiovascular system and nervous system.

## Data availability statement

The raw data supporting the conclusions of this article will be made available from the corresponding author by appropriate request.

## Author contributions

XX designed the study, wrote the manuscript, and completed the revision. HD, PL, JS, and JT analyzed the data. All authors contributed to the article and approved the submitted version.
